# Older Breast Cancer Survivors: Perspectives on Healthcare Encounters and Unmet Needs

**DOI:** 10.1093/geroni/igab046.1397

**Published:** 2021-12-17

**Authors:** Victoria Raveis, Anita Nirenberg, Yumeng Liu, Simona Kwon

**Affiliations:** 1 New York University, New York University, New York, United States; 2 Hunter College, Hunter College, CUNY, New York, United States; 3 New York University, New York, New York, United States; 4 NYU School of Medicine, New York University School of Medicine, New York, United States

## Abstract

Breast cancer treatment advances have lengthened the survivorship period. Limited attention has focused on the myriad issues breast cancer survivors experience related to their cancer and other health conditions as they age. Focus groups, conducted Fall 2019 – Spring 2020 with a diverse sample of breast cancer survivors from the New York metropolitan region (N=28) explored survivors’ healthcare encounters and goals, quality of life, survivorship lifestyle, other health conditions and risks, e.g. emergence of COVID-19. Participants were 40-82 years old (57% were 56 or older); racially diverse (57% White, 18% Black, 14% Hispanic, 11% Bi-racial); 32% were married/partnered and 57% were parents. Mean diagnosis age was 51. Treatments received included lumpectomy (64%), chemotherapy (57%), radiation (46%), hormonal therapy (39%), and single/bilateral mastectomy (36%). Survivors expressed the importance of restoring normality in their life and the necessity to be pro-active in ensuring their health issues were addressed in medical encounters. Person-centered care and clinician engagement was valued, but not routinely experienced. Survivors evaluated treatment options not just on being cancer-free, but on how it would impact their whole life. They expressed concerns about the future and anxiety over long-term survival. Long term survivors, recipients of early experimental and/or extensive treatments, worried about an emergence of long-delayed adverse health consequences and complications managing other health issues in the future, particularly as they grew older. COVID-19 raised additional health concerns, particularly among those with high risk health conditions due to prior cancer treatments; various self-mandated protective activities were integrated into their self-care practices.

